# End-of-life experience for older adults in Ireland: results from the Irish longitudinal study on ageing (TILDA)

**DOI:** 10.1186/s12913-020-4978-0

**Published:** 2020-02-14

**Authors:** Peter May, Lorna Roe, Christine A. McGarrigle, Rose Anne Kenny, Charles Normand

**Affiliations:** 1 Centre for Health Policy & Management, Trinity College Dublin, 3-4 Foster Place, Dublin 2, Ireland; 2 The Irish Longitudinal study on Ageing (TILDA), Trinity College Dublin, Lincoln Gate, Dublin 2, Ireland; 3 Mercer’s Institute for Successful Ageing, St James’s Hospital, Dublin 8, Ireland; 4 Cicely Saunders Institute for Palliative Care, Rehabilitation and Policy, King’s College London, Bessemer Road, London, SE5 9PJ UK

**Keywords:** End of life, Ageing, Longitudinal study, Hospital, Hospice, Cancer, Utilisation, Policy

## Abstract

**Background:**

End-of-life experience is a subject of significant policy interest. National longitudinal studies offer valuable opportunities to examine individual-level experiences. Ireland is an international leader in palliative and end-of-life care rankings. We aimed to describe the prevalence of modifiable problems (pain, falls, depression) in Ireland, and to evaluate associations with place of death, healthcare utilisation, and formal and informal costs in the last year of life.

**Methods:**

The Irish Longitudinal Study on Ageing (TILDA) is a nationally representative sample of over-50-year-olds, recruited in Wave 1 (2009–2010) and participating in biannual assessment. In the event of a participant’s death, TILDA approaches a close relative or friend to complete a voluntary interview on end-of-life experience. We evaluated associations using multinomial logistic regression for place of death, ordinary least squares for utilisation, and generalised linear models for costs. We identified 14 independent variables for regressions from a rich set of potential predictors. Of 516 confirmed deaths between Waves 1 and 3, the analytic sample contained 375 (73%) decedents for whom proxies completed an interview.

**Results:**

There was high prevalence of modifiable problems pain (50%), depression (45%) and falls (41%). Those with a cancer diagnosis were more likely to die at home (relative risk ratio: 2.5; 95% CI: 1.3–4.8) or in an inpatient hospice (10.2; 2.7–39.2) than those without. Place of death and patterns of health care use were determined not only by clinical need, but other factors including age and household structure. Unpaid care accounted for 37% of all care received but access to this care, as well as place of death, may be adversely affected by living alone or in a rural area. Deficits in unpaid care are not balanced by higher formal care use.

**Conclusions:**

Despite Ireland’s well-established palliative care services, clinical need is not the sole determinant of end-of-life experience. Cancer diagnosis and access to family supports were additional key determinants. Future policy reforms should revisit persistent inequities by diagnosis, which may be mitigated through comprehensive geriatric assessment in hospitals. Further consideration of policies to support unpaid carers is also warranted.

## Background

End-of-life healthcare use is a subject of significant policy interest. People living and dying with serious medical illness experience poor outcomes and high costs in systems originally designed to provide acute, episodic care [1], and are growing in number due to demographic change [2]. The burden of poor experience falls disproportionately on those of low socioeconomic status [3] and equity gaps are also growing as the population ages [4]. Research studies to inform improvement efforts face challenges [5].

International rankings of end-of-life care show a wide variation in palliative care capacity across countries [6, 7]. Such comparisons are useful but have important limitations. They use routine administrative data on macro-level supply, but do not capture the needs and experiences of families, or determinants of outcomes [8, 9]. They also do not measure unpaid family supports near end of life, which may be of a similar magnitude to formal utilisation [10–13]. Rankings must be interpreted carefully as high capacity relative to other health care systems does not necessarily correspond to adequate and equitable supply within that country.

Ireland is ranked fourth worldwide for end-of-life care, meaning that there is relatively high per capita palliative care provision at the population level [6, 7]. Corresponding individual-level data on end-of-life experience in Ireland has never previously been available. The Irish Longitudinal Study on Ageing (TILDA) is a representative sample of community-dwelling over-50-year-olds, who were recruited in Wave 1 (2009–2010) and participate via ongoing assessment [14]. In the event of a participant death, TILDA approaches a family member or close friend to complete a voluntary interview on end-of-life experience. This provides a rare opportunity to interrogate high-quality population-level end-of-life data in a country with well-established palliative care provision [15, 16].

### Objectives

To describe prevalence of modifiable problems (pain, falls, depression) and to analyse determinants of place of death, healthcare utilisation, and formal and informal costs in the last year of life, among older adults in Ireland.

## Methods

### Participants

TILDA participants confirmed as having died between Waves 1 to 3 (2010–2014), and for whom an end-of-life interview was completed.

### Study design

TILDA is a prospective nationally representative study of community dwelling adults in the Republic of Ireland aged 50 years and over [17, 18]. TILDA participants were selected using multi-stage, stratified random sampling that identified 640 geographical areas, truncated by socio-economic characteristics, and selected households within each area [19]. Details of sample maintenance are available elsewhere [20].

TILDA collects information on a wide range of topics including health, economic, social and family circumstances. Wave 1 was conducted between 2009 and 2011, with each participant completing a computer-assisted personal interviews (CAPI) and self-completion questionnaires (SCQ), and a comprehensive health assessment by a trained nurse [14, 21, 22]. Subsequent waves are biannual (Wave 2 in 2012, Wave 3 in 2014, etc). CAPI and SCQ follow-up occurs at each Wave; health assessments were conducted at Wave 3 and are planned again at Wave 6 in 2020.

Details on how participant deaths are identified and how family and friends are approached for the end-of-life interview are provided in Appendix Part 1. The end-of-life interview covers demographics; disability and level of assistance; physical, behavioural and mental health; and utilisation and assets; and complements equivalent sections of regular TILDA participant interviews [23]. This study employs regular Waves 1 and 2, and all end-of-life interviews completed on the behalf of a participant who died between Waves 1 and 3.

Ethical approval for each wave of TILDA is obtained from the Faculty of Health Sciences Research Ethics Committee in Trinity College Dublin. Participants are provided with sufficient information to make an informed decision about their participation including advance notice of the study. Written consent is obtained for separate components of the study (i.e. interview, health assessment, blood samples); participants may refuse to take part in or withdraw at any time without justification. TILDA data collection involves minimal risk, invasion, burden and discomfort, though there are potential ethical issues that may arise, e.g. excessive time commitment or distress due to the nature of questions being asked, immediate and/or unforeseen medical concerns. Procedures are in place to address these issues (summarised in Appendix Part 1) [20]. The collection of end-of-life data is subject to particular care; an appropriate length of time is left before contacting next-of-kin.

### Setting

Ireland has a relatively young population among high-income countries but faces the same demographic ageing and falling workforce participation [24]. The Irish healthcare system has mixed public and private provision. A means-tested medical card confers free primary and hospital care as well as limited co-payments for prescriptions [24]. Those without a medical card contribute capped co-payments for hospital care and prescriptions, and pay full primary care costs out of pocket. Private insurance to access privately provided hospital care is bought by 53% of over 50s in the context of lengthy waiting lists for planned care [25]. International comparisons show unusually high acute hospital bed occupancy and relatively low primary and community care provision [26].

In 2001 Ireland was among the first countries to establish a national policy of universal palliative care access on the basis of need, integrated in care of all life-limiting illnesses [27]. Access to services increased substantially in the 7 years following policy implementation but funding remained about 60% of that required to meet universal provision [28]. Access was in 2001 characterised by regional inequity – the large cities had established services, while many rural areas had no history of provision – but new funding was not targeted at low-provision areas and inequities persisted [28, 29]. Service expansion in the last decade has been much more modest due to the 2008 global financial crash and its aftermath, which had severe fiscal ramifications for Ireland’s health system [30]. The most recent data suggest that still all regions have insufficient funding to achieve universal provision, and that level of access reflects geographic location not individual need [31]. Policymakers plan an evaluation and revision of the national policy in 2020–2021 [32].

### Variables

#### Outcomes of interest

In describing modifiable problems that are markers of poor experience, we identified three variables of interest: regular pain, regular depression and falls towards the end of life.

For regression analyses evaluating association between predictors and outcome, we identified three domains of interest: place of death (=hospital, own home, nursing home, inpatient hospice), healthcare utilisation (hospital inpatient stays, outpatient visits and emergency department (ED) admissions; general practitioner (GP) visits; home help supports), and formal and informal costs in the last year of life [33, 34].

Definition and calculation of these outcomes are detailed in Appendix Part 2.

#### Explanatory variables

To identify explanatory variables we drew on Andersen’s model of predisposing, enabling, need characteristics; and prior utilisation [35]. Our final model included 14 baseline predictors, summarised in Table 1. Definition and calculation of these predictors, and diagnostic appraisal of the model, are detailed in Appendix Part 2.

**Table 1:** Decedent characteristics (*n* = 375)

		%	N
**Gender**	*Female*	46%	171
**Living alone**	*Yes*	44%	166
**Education (highest achieved)**	*Tertiary/higher*	15%	57
**Location of residence**	*A rural area*	47%	156
**Medical or GP card**	*Yes*	87%	326
**Private health insurance**	*Yes*	37%	139
**Diagnosis of**	*Heart disease*	55%	205
	*Cancer*	46%	174
	*Dementia*	13%	48
**Short illness**	*Yes*	20%	75
**Frailty**	*Yes*	38%	141
		**Mean**	**(Range)**
**Age**	*Years*	77.7	(71–86)
**ADL**	*Total (/6)*	2.4	(0–6)
**Chronic conditions**	*Total (/17)*	2.9	(2–4)

Range: 25th% to 75th%. All variables measured at death via end-of-life interview except ‘Education’, which was recorded at first TILDA interview; ‘Location’ and ‘Frailty’, which were drawn from the last TILDA interview by the decedent; and diagnosis/total of serious chronic conditions, which were collated from all participant Waves and end-of-life interview

**Definitions**

Medical Cards provide free access to a GP, community health services, dental services, prescription medicine costs, hospital care and other benefits; access is provided on the basis of means and age; 38% of the population have a medical card including 89% of people over 70 years [36]. ‘Diagnosis of’: binary variable = 1 if in either the participant’s Wave 1 or Wave 2 interview, or the end-of-life interview completed by a family member or friend, a doctor had told the participant that they had this condition. ‘Heart disease’: any one of angina, heart attack, congestive heart failure (CHF), stroke; ‘Dementia’: any one of dementia, Alzheimer’s, serious memory impairment; ‘Short Illness’: Was the person ill for a week or less prior to death?; ‘Frailty’: A binary variable was generated indicating the presence of frailty as a FI score greater than 0.25; ‘ADL’: Activities of Daily Living. How many of the following six activities did the person who died require help with in the last 3 months of life: dressing, crossing a room, bathing, eating, getting in/out of bed, using the toilet; ‘Chronic conditions’: total number of serious chronic conditions, using absence/presence (0|1) of angina, heart attack, CHF, stroke, cardiac arrhythmia, hypertension, diabetes, chronic lung disease, cancer, Parkinson’s, psychiatric problems including depression or anxiety, alcohol or drug abuse, dementia, stomach ulcers, cirrhosis or serious liver damage, thyroid problems, kidney damage

**Reference cases**

Living alone: Living with others; Education: Primary or secondary; Location of residence: Dublin, urban or peri-urban

#### Missing data

“Don’t know” is an available response throughout the end-of-life interview. There were few missing data on explanatory variables in the end-of-life interviews and replacement data were sourced from the regular TILDA waves. E.g. where a respondent did not know the participant’s age at death, this could be calculated using date of birth from Wave 1 and date of death.

Missing outcomes data can be observed in Table 3. Where the respondent replied “don’t know” for a utilisation category determined by a discrete outcome (inpatient, outpatient, ED and GP visits) we excluded the decedent from analysis. Where the respondent replied “don’t know” for one component of a composite outcome of interest (receipt of formal home supports, formal costs, informal costs), we imputed the median value from the rest of the end-of-life sample. E.g. if a respondent did not know how many times a decedent had visited the GP, the decedent’s GP costs are estimated as the median among non-missing responses. All missing data reflect the end-of-life interviewee not knowing the answer or refusing to answer a given question. Therefore, these data are missing not at random.

To preserve anonymity TILDA does not report cell sizes smaller than 20; all places of death with fewer than 20 were excluded from primary analysis.

### Statistical methods

Prevalence of modifiable problems are reported descriptively. All other outcomes of interest are analysed using multivariate regression: outcomes of interest were regressed against all explanatory factors listed in Table 1 and *p* < 0.05 was taken as significant. For place of death, a nominal variable, we used multinomial logistic regression with hospital death as the base case, calculating relative risk ratios (RRR) and a 95% confidence interval. For frequency utilisation data (inpatient, outpatient, ED and GP visits) and costs, we used ordinary least squares (OLS) regression on the square-root transformed outcome of interest. This model was chosen after comparative evaluation of linear and nonlinear alternatives [37–40]. For one binary utilisation outcome (receipt of formal home supports), we used logistic regression.

## Results

### Participants and descriptive data

Of 516 confirmed deaths between Waves 1 and 3, 375 (73%) end-of-life interviews have been completed (Appendix Part 3). The majority were male (54%) with an average age at death of 77.7 (Table 1). Substantial differences in functional capacity are observable: approximately a third of people who died did not need any assistance with activities of daily living (ADLs) whereas another third needed assistance with all six.

A comparison on independent variables of decedents with and without an end-of-life interview found one significant difference: decedents with an interview were significantly older at recruitment than those without (Appendix Part 4). Compared to population-level statistics, deaths in TILDA were slightly younger and with more deaths from cancer (Appendix Part 5).

### Outcome data

Prevalence of modifiable problems is presented in Table 2. A significant proportion of decedents were regularly troubled by pain (50%) and depression (45%) in the last year of life, and/or experienced a fall (41%) in the last 2 years.

**Table 2:** Prevalence of modifiable problems near end of life

	%	N
**Often troubled by pain**	50	187
**Frequently/sometimes troubled by depression**	45	169
**Experienced a fall**	41	154

Summary data on place of death and health care use are presented in Table 3.

**Table 3:** Place of death and healthcare use in the last year of life

Place of death	N	%		
**Hospital**	172	46%		
**Home**	100	27%		
**Hospice**	43	11%		
**Nursing home**	39	10%		
**Other**	21	6%		
**Total**	375	100%		
**Utilisation (Frequency)**	**N**	**Mean**	**Median**	**SD**
**Inpatient admissions**	374	2.1	1	4.3
**Outpatient visits**	366	5.0	1	10.7
**ED visits**	372	1.3	0	2.8
**GP visits**	350	10.2	6	12.5
	**N**	**%**		
**Home care (Yes)**	375	57%		
**Costs (€)**	**N**	**Mean**	**Median**	**SD**
**Formal costs**	375	33,129	21,821	37,892
**Informal costs**	375	19,748	6192	31,499

*SD* Standard deviation

*ED* Emergency department, *GP* General Practitioner. For inpatient, outpatient, ED and GP there were a small number (1–25) of end-of-life interview respondents who did not know or refused to answer. These are excluded from the sample in each case

Receipt of formal home support: at least one of home help, meals on wheels, personal care attendant and/or public health nurse

Of 375 decedents, 172 (46%) died in hospital, 100 (27%) at home, 43 (11%) in a hospice, 39 (10%) in a nursing or residential home, and 21 (6%) in other places.

Frequency data show a mean of 2.1 inpatient admissions, 5.0 outpatient admissions, 1.3 ED visits and 10.2 GP visits. These outcomes had missing data (indicating that the respondent either did not know or refused to answer) in the range 1–25 (0.2–6%). Over half of participants received home supports (57%).

Mean estimated formal health and social care costs in the last year of life were €33,129 and mean estimated informal costs were €19,748, meaning that informal costs account for 37% of all costs. Median use of informal care (€6192) was equivalent to approximately 1 h per day. In all utilisation and cost categories, a minority accounted disproportionately for use.

### Main results

Associations with place of death are presented in Table 4, expressed as RRRs. There were three significant associations for a home death versus hospital: people living alone were half as likely to die at home as those who lived with others (RRR: 0.54; 95%CI: 0.31 to 0.94), and people with a cancer diagnosis (2.49; 1.29 to 4.81) or who died following a short illness (4.06; 1.91 to 8.62) were more likely to die at home. There were three significant associations for a hospice death versus hospital: cancer diagnosis (10.20; 2.66 to 39.15) and higher numbers of chronic conditions (1.42; 1.05 to 1.94) had a positive correlation with hospice; heart disease diagnosis (0.27; 0.09 to 0.77) had a negative correlation. There were five significant associations for a nursing home death versus hospital: older age (1.08; 1.02 to 1.15), higher ADL total (1.52; 1.24 to 1.86) and dementia (3.68; 1.27 to 10.66) were positively correlated with nursing home; cancer diagnosis (0.21; 0.06 to 0.74) and rural residency (0.33; 0.13 to 0.81) were negatively correlated.

**Table 4:** Associations between decedent characteristics and place of death (versus hospital) (*n* = 354)

Base: Hospital death (*n* = 172)		Home death (*n* = 100)			Inpatient hospice death (*n* = 43)			Nursing Home death (*n* = 39)		
		RRR	p	95% CI	RRR	p	95% CI	RRR	p	95% CI
**Age**	*Years*	1.00	0.83	0.97 - 1.03	0.99	0.72	0.95 - 1.04	**1.08**	**0.01**	**1.02–1.15**
**Gender**	*Female*	0.69	0.18	0.40 - 1.19	0.67	0.33	0.30 - 1.49	0.53	0.17	0.21–1.30
**Living alone**	*Yes*	**0.54**	**0.03**	**0.31** - **0.94**	0.63	0.27	0.28 - 1.43	1.94	0.17	0.76–4.95
**Education**	*Tertiary/higher*	1.12	0.77	0.52 - 2.44	1.73	0.27	0.65 - 4.60	0.30	0.23	0.04–2.17
**Location**	*Rural*	1.06	0.82	0.62 - 1.82	0.42	0.05	0.17 - 1.00	**0.33**	**0.02**	**0.13–0.81**
**Medical/GP card**	*Yes*	1.82	0.18	0.77 - 4.34	1.85	0.33	0.54 - 6.29	1.09	0.94	0.11–11.27
**Insurance**	*Yes*	1.31	0.39	0.71 - 2.41	1.85	0.17	0.77 - 4.43	1.31	0.60	0.48–3.56
	*Total*	1.06	0.34	0.94 - 1.18	1.16	0.06	1.00 - 1.35	**1.52**	**< 0.005**	**1.24–1.86**
**Diagnosis of**	*Heart disease*	1.38	0.35	0.71 - 2.69	**0.27**	**0.01**	**0.09 - 0.77**	0.60	0.33	0.21–1.70
	*Cancer*	**2.49**	**0.01**	**1.29** - **4.81**	**10.20**	**< 0.005**	**2.66 - 39.15**	**0.21**	**0.02**	**0.06–0.74**
	*Dementia*	1.27	0.63	0.47 - 3.42	0.84	0.85	0.14 - 5.13	**3.68**	**0.02**	**1.27–10.66**
**Chronic conditions**	*Total*	0.89	0.27	0.73 - 1.09	**1.42**	**0.02**	**1.05 - 1.94**	1.10	0.54	0.80–1.52
**Short illness**	*Yes*	**4.06**	**< 0.005**	**1.91** - **8.62**	0.75	0.80	0.08 - 6.84	0.39	0.28	0.07–2.15
**Frailty**	*Yes*	1.13	0.72	0.58 - 2.18	1.01	0.98	0.38 - 2.73	0.48	0.15	0.18–1.29

For variables legend, see Table 1. *p p* value, *RRR* Relative risk ratio, *95% CI* confidence interval. Statistically significant (*p* < 0.05) highlighted bold

Corresponding associations with utilisation are presented in Table 5, expressed as OLS coefficients. ADL total was associated with higher inpatient admissions (coefficient: 0.06; 95% CI: 0.02 to 0.10); dementia (− 0.34; − 0.66 to − 0.02) and short illness (− 0.62; − 0.90 to − 0.33) with fewer inpatient admissions. Cancer diagnosis (0.60; 0.18 to 1.01) and total chronic conditions (0.15; 0.03 to 0.27) were positively correlated with outpatient utilisation, and frailty (− 0.50; − 0.91 to − 0.10), dementia (− 0.62; − 1.17 to − 0.07), short illness (− 0.63; − 1.12 to − 0.14) and age (− 0.03; − 0.05 to − 0.01) were associated with lower outpatient utilisation. Total number of ADLs (0.06; 0.02 to 0.09) was positively correlated with ED admissions, and short illness (− 0.47; − 0.72 to − 0.21) and rural residence (− 0.19; − 0.37 to − 0.02) were negatively associated. Living alone (0.47; 0.11 to 0.83) was positively associated with GP visits and short illness (− 0.66; − 1.16 to − 0.16) was negatively associated. Having a medical card (1.03; 0.23 to 1.84), ADL total (0.12; 0.02 to 0.21), cancer (0.65; 0.07 to 1.24) and frailty (0.86; 0.29 to 1.43) were positively associated with home help receipt, and short illness (− 1.46; − 2.16 to − 0.75) was negatively associated.

**Table 5:** Associations between decedent characteristics and healthcare utilisation in the last year of life

		Inpatient admissions (*n* = 374)		Outpatient clinic visits (*n* = 366)		ED visits (*n* = 372)		GP visits (*n* = 350)		Home care use (*n* = 375)	
		Coeff.	95% CI	Coeff.	95% CI	Coeff.	95% CI	Coeff.	95% CI	Coeff.	95% CI
**Age**	*Years*	0.00	− 0.01 - 0.01	**− 0.03**	**− 0.05 - − 0.01**	-0.01	− 0.02 - 0.00	− 0.01	− 0.03 - 0.01	0.02	− 0.01 - 0.05
**Gender**	*Female*	0.10	− 0.10 - 0.30	0.16	− 0.17 - 0.50	0.06	− 0.11 - 0.24	0.25	− 0.10 - 0.60	0.00	− 0.48 - 0.47
**Living alone**	*Yes*	−0.05	−0.25 - 0.15	− 0.23	− 0.58 - 0.11	0.07	− 0.11 - 0.25	**0.47**	**0.11–0.83**	0.18	− 0.30 - 0.67
**Education**	*Tertiary/higher*	− 0.13	− 0.41 - 0.16	0.17	− 0.33 - 0.67	− 0.18	−0.44 - 0.08	− 0.07	−0.57 - 0.43	− 0.49	− 1.18 - 0.20
**Location**	*Rural*	0.00	−0.20 - 0.20	−0.22	− 0.56 - 0.11	**−0.19**	**− 0.37 - -0.02**	0.10	− 0.25 - 0.44	0.31	− 0.16 - 0.79
**Medical/GP card**	*Yes*	0.08	−0.24 - 0.40	0.27	−0.28 - 0.83	0.17	−0.12 - 0.45	0.54	−0.02 - 1.10	**1.03**	**0.23–1.84**
**Insurance**	*Yes*	0.00	−0.22 - 0.23	−0.18	− 0.56 - 0.20	−0.04	− 0.24 - 0.16	0.20	− 0.19 - 0.59	0.32	− 0.22 - 0.86
**ADL**	*Total*	**0.06**	**0.02–0.10**	0.04	−0.02 - 0.11	**0.06**	**0.02–0.09**	− 0.03	−0.10 - 0.04	**0.12**	**0.02–0.21**
**Diagnosis of**	*Heart disease*	−0.01	−0.25 - 0.23	− 0.15	−0.57 - 0.27	0.09	−0.13 - 0.31	0.06	−0.37 - 0.49	0.40	−0.20 - 0.99
	*Cancer*	0.11	−0.13 - 0.35	**0.60**	**0.18–1.01**	− 0.08	−0.29 - 0.13	− 0.02	−0.44 - 0.40	**0.65**	**0.07–1.24**
	*Dementia*	**−0.34**	**−0.66 - -0.02**	**− 0.62**	**− 1.17 - -0.07**	−0.25	− 0.54 - 0.04	0.11	− 0.45 - 0.67	0.05	− 0.71 - 0.81
**Chronic conditions**	*Total*	0.03	−0.04 - 0.10	**0.15**	**0.03–0.27**	0.03	−0.03 - 0.09	0.08	−0.04 - 0.21	− 0.15	−0.32 - 0.02
**Short illness**	*Yes*	**−0.62**	**−0.90 - -0.33**	**− 0.63**	**− 1.12 - -0.14**	**−0.47**	**− 0.72 - -0.21**	**−0.66**	**− 1.16 - -0.16**	**− 1.46**	**− 2.16 - -0.75**
**Frailty**	*Yes*	− 0.04	− 0.28 - 0.19	**− 0.50**	**−0.91 - -0.10**	0.18	−0.03 - 0.39	0.29	−0.12 - 0.70	**0.86**	**0.29–1.43**

For variables legend, see Table 1. 95% CI: confidence interval. Statistically significant (*p* < 0.05) highlighted bold

Associations for healthcare costs are presented in Table 6, expressed as OLS coefficients. ADL total (10; 7 to 13) was positively correlated with higher formal and informal costs; short illness (− 45; − 67 to − 24) was negatively correlated. Older age (2; 1 to 3) and frailty (21; 0 to 42) were associated with higher informal costs; short illness (− 29; − 55 to − 4), living alone (− 42; − 60 to − 24) and living in a rural area (− 20; − 37 to − 2) were each associated with lower informal costs.

**Table 6:** Associations between decedent characteristics and health and social care costs in the last year of life (*n* = 375)

		Formal			Informal		
		Coeff.	p	95% CI	Coeff.	p	95% CI
**Age**	*Years*	0	0.51	−1 - 1	**2**	**< 0.005**	**1–3**
**Gender**	*Female*	12	0.12	−3 – 27	9	0.34	−9 – 26
**Living alone**	*Yes*	3	0.69	−12 – 18	**−42**	**< 0.005**	**− 60 - -24**
**Education**	*Tertiary/higher*	1	0.91	−20 – 23	−18	0.17	−43 – 8
**Location**	*Rural*	−14	0.05	−29 – 0	**−20**	**0.03**	**−37 - -2**
**Medical/GP card**	*Yes*	8	0.52	−16 – 32	−1	0.93	−30 – 27
**Insurance**	*Yes*	−7	0.39	−24 – 9	6	0.53	−13 – 26
**ADL**	*Total*	**10**	**< 0.005**	**7–13**	**15**	**< 0.005**	**12–19**
**Diagnosis of**	*Heart disease*	−2	0.81	−20 – 16	−4	0.68	−26 – 17
	*Cancer*	14	0.12	−4 – 32	16	0.13	−5 – 37
	*Dementia*	−1	0.96	−24 – 23	− 13	0.38	−41 – 16
**Chronic conditions**	*Total*	3	0.27	−2 – 8	0	0.94	−6 – 6
**Short illness**	*Yes*	**−45**	**< 0.005**	**−67 - -24**	**−29**	**0.03**	**−55 - -4**
**Frailty**	*Yes*	8	0.39	−10 - 25	**21**	**0.05**	**0–42**

For variables legend, see Table 1. *p* p value, *95% CI* confidence interval. Statistically significant (*p* < 0.05) highlighted bold

### Sensitivity analyses

We re-ran the place of death regression first without those variables with low cell count (*N* < 10 for any place of death) and second using prior utilisation variables as additional predictors. Our results were not substantively different (Appendix Part 6).

In addition to multiple *ex ante* model diagnostics for the utilisation analyses, we checked our reported results using generalised linear models: (Poisson, log) for utilisation and (gamma, log) for costs. Our results were not substantively different.

We checked that our results were not sensitive to time dimensions in two ways. We regressed year of interview on our primary outcome, place of death, to see if policy or system change over time may be driving results. And we regressed time from date of death to the end-of-life interview on costs to see if response times or interviewee memory may be driving results. There were no significant associations.

## Discussion

### Key results

A significant proportion of decedents were regularly troubled by pain, depression and falling near end of life. Place of death among older people in Ireland appears determined by a combination of predisposing, enabling and need factors. A home death, typically considered the desirable outcome all else being equal, [41, 42] was correlated with cancer diagnosis and short illness while those who lived alone were less likely to die at home. Hospice deaths were heavily driven by cancer diagnosis and disease burden while nursing home decedents tended to be older with higher functional disability and more non-cancer diagnoses including dementia.

Utilisation and costs were driven most heavily by need characteristics. ADL total was consistently associated with higher utilisation and costs. A short illness prior to death was associated with lower use of most services. However, some predisposing and enabling factors were also important. Notably living alone and living in a rural area were both associated with significantly lower informal care costs, but this was not matched with a corresponding increase in formal care, suggesting the potential for those groups to be underserved. Previous research has shown that end-of-life care in Ireland is characterised by an urban-rural divide, [28] and international studies have also noted equivalent differences [43–46]. Consistent with prior work, old age was not found to be associated with higher service use, although it was associated with more informal care [25, 47].

### Interpretation and policy implications

Ireland is near the top of international rankings for end-of-life care provision. Nevertheless, significant gaps and difficulties in end-of-life experience for older people are observable. Pain, depression and falls are treatable difficulties. High prevalence indicates inadequate identification and management. These problems, as well as inequities in place of death by diagnosis, could be mitigated through improved geriatric assessment in hospitals and general practice.

Hospital deaths, typically considered an undesirable outcome all else being equal, are high in Ireland by international standards. This statistic, and strong association with living alone and diseases other than cancer, indicates inadequate community and home care supports. Increased home care services would reduce reliance on acute hospital stays and hospital deaths [26, 48].

The associations between serious illness, disability burden and long illness prior to death and higher utilisation are not surprising but nevertheless reinforce the widely observed imperative that systems must be reformed from a focus on acute, episodic care to the appropriate treatment and support of chronic long-term disease [7, 49–51].

Our results also follow multiple prior studies emphasising the importance of informal care in understanding and measuring end-of-life experience [13, 52] – in our study, unpaid help constitutes over a third of all care received, and living with others appears to be associated both with home death and more overall care. Greater consideration must be given as to how policy can materially support informal care in people with serious and terminal illness. It is important that those at risk of isolation living alone and/or in rural areas are identified and receive additional supports from the formal system to substitute for the informal care that is less available to them.

The deaths in this study occurred between ten and 14 years after implementation of a national policy recommending that palliative care be available as a component of all chronic disease management since 2001. Routine statutory data collection has previously revealed significant gaps in provision. Policy analysis has emphasised that a well-established national policy is insufficient to meet population health needs without additional resources, implementation and staff capacity. Individual-level TILDA data offer additional perspective on uneven end-of-life experience. That cancer patients are more likely to die at home or in hospice indicates that universal provision irrespective of diagnosis remains an unfulfilled policy ambition.

### Strengths and limitations

The main strength of this study is a rich, population-representative dataset. The results therefore provide an opportunity to interrogate end-of-life experience, including unpaid informal care, against a wide set of characteristics. With respect to generalisability, Ireland is a country with well-established palliative care services and policies and so the results will be of interest internationally as other countries update and expand their own provision.

There are nevertheless a number of limitations. First, initial recruitment of community-dwelling people means that the end-of-life sample is yet to reach full maturity, being on average a little younger than the general population. Second, the sample is heterogeneous with broadly one third healthy and active until death, and another third with high functional disability, and it is possible that larger future samples will show more distinct classes with different determinants of outcome (Appendix 7). Third, we used OLS regression on square-root-transformed utilisation outcomes, which delivers coefficients that are hard to interpret but cannot be reliably retransformed [53]. This model was chosen after extensive evaluation of linear and nonlinear alternatives [40]. Fourth, proxy interviews may misrepresent participant experience [54]. Fifth, informal costs are calculated derived only from unpaid help provided in performing (I) ADLs and not any other activities, and required the combination of participant-reported answers and end-of-life interview.

Finally, this is a decedent cohort study where death is an eligibility criterion for participants. This study design has well-documented strengths, notably population representativeness, [8] and limitations, particularly for causal inference [55]. Forward-counting analyses that enrol people from diagnosis could evaluate quality-of-life outcomes such as pain and depression alongside costs in a full economic evaluation, and disentangle the associations between household structure, formal care use and informal care. Such analyses are planned.

All end-of-life studies have to be conducted within these and similar constraints, [8, 55–57] and our specific limitations are offset by the strength and richness of the data.

## Conclusion

Ireland is ranked among the leading countries for end-of-life care provision yet significant gaps and difficulties in experience for older people are observable. People with cancer are more likely to die at home or in hospice, contrary to policies recommending palliative care as a component of all serious chronic disease management. High proportion of hospital deaths, including strong association with living alone and diseases other than cancer, indicates inadequate community and home care supports. Unpaid informal care accounts for 37% of all care received but access to this care, as well as important outcomes, may be adversely affected by living alone or in a rural area. Further consideration of policies to support unpaid carers, is warranted.

### Supplementary information

**Additional file 1.**

## Figures and Tables

**Fig. 1:**
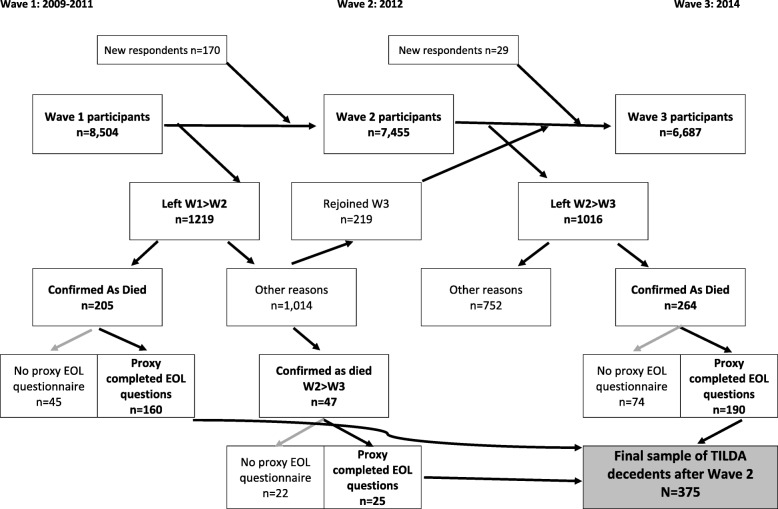
How the analytic sample was reached

## Data Availability

The Exit Interview data used in this analysis are not currently available due to privacy concerns with the small end-of-life sample. Researchers interested in using regular waves of TILDA data may access the data for free from the following sites: Irish Social Science Data Archive (ISSDA) at University College Dublin http://www.ucd.ie/issda/data/tilda/; Interuniversity Consortium for Political and Social Research (ICPSR) at the University of Michigan http://www.icpsr.umich.edu/icpsrweb/NACDA/studies/34315.

## Abbreviations

ADL: Activity of daily living
CAPI: Computer-assisted personal interviews
CHF: Congestive heart failure
CI: Confidence interval
ED: Emergency department
GP: General practitioner
IADL: Instrumental activity of daily living
OLS: Ordinary least squares
RRR: Relative risk ratios
SCQ: Self-completion questionnaires
SD: Standard deviation
TILDA: The Irish Longitudinal Study on Ageing
