# Supplementary material for: End-of-life experience for older adults in Ireland: results from the Irish longitudinal study on ageing (TILDA)
Source: BMC Health Serv Res. 2020 Feb 14;20:118. doi: 10.1186/s12913-020-4978-0 (PMC7023768; doi:10.1186/s12913-020-4978-0)
Supplement: Supplementary file 1 — Additional file 1. [file 12913_2020_4978_MOESM1_ESM.docx]

## Appendix

### 1 Interview procedures (see also ^1^)

#### CAPI interviews

Topic-specific training is provided for all interviewers since they ask questions of a potentially sensitive nature. Training on how to handle distressed respondents is given to all interviewers by the core research team. An emergency contact number for the fieldwork agency is made available to all interviewers working on the study. This is used if unanticipated problems concerning their own, or the respondent’s welfare emerge in the course of the interview. In addition, a telephone number is included on the respondent information leaflet for respondents to contact TILDA if required. The person answering the call is trained to respond sympathetically to any callers and to direct their query to the appropriate person in the study team (for example, the Project Manager).

The most recent interviewer feedback and debriefing (Wave 5) have not uncovered any areas of the main or proxy questionnaire which caused distress.

#### End-of-life interviews

In the event that a respondent has died, TILDA seeks to conduct an end-of-life interview with a friend or relative who has close knowledge of the deceased. The deaths of TILDA participants are identified in different ways. The study itself or the interviewer who has arranged to conduct a CAPI may be contacted in advance of that interview. The interviewer may find out when they go to conduct the interview. Linkage to statutory mortality records ensures that in the long run we identify all deaths for participants within Ireland.

Usually, end-of-life interviews are sought from a spouse or family member (e.g. when they confirm the participant has passed away); can vary depending on who is willing to provide the interview. If no family member, it could be a carer or friend but it should be some-one who is in a position to be able to provide info about the participant’s last months of life. TILDA protocol is not to seek an interview within first six months of death.

There is a risk that being asked questions about their deceased loved one will renew emotions resulting from their grief and loss which could lead to the respondent becoming distressed. All interviewers are trained in how to conduct the ‘end of life’ interviews, including different strategies to prevent and overcome any distress resulting from the interview. These include taking extra time to explain the need for the interview, reacting sympathetically and empathically to emotions and clarifying the interviewee’s understanding of the study.

In addition, reminders of their right not to answer specific questions if they would prefer not to are highlighted throughout the interview, particularly if the person becomes distressed. While we recognise that in some cases, individuals may be more likely to decline or drop out as a result of these recommendations, we believe that this is a necessary limitation in conducting research with the family and friends of a deceased respondent and the higher mandate is to protect the interviewee and minimise burden.

Similar questionnaires about similar experiences have been used in international studies, for example, the Health and Retirement Survey (HRS) in the US and the English Longitudinal Study of Ageing (ELSA). The resulting data has been shown to be enormously useful in developing our understanding of end-of-life issues.

The length of the questionnaire may cause inconvenience to the participant. If the respondent finds that the questionnaire is too long, the researcher will offer to take a break in the administration or to return to complete the questionnaire on another occasion. An average end-of-life interview takes about 45 minutes.

### 2 How variables were defined

#### Outcomes of interest

In describing modifiable problems that are markers of poor experience, we identified three variables of interest: regular pain [“Was [the person] often troubled with pain in the last year of life?”; Yes/No/Don’t know/Refuse], regular depression [Do you think [he/she] was depressed during [his/her] last year of life?; Yes frequently/Yes, sometimes/No/Don’t know/Refuse] and falls [“Had [the person] person fallen down in the last two years of life?”; Yes/No/Don’t know/Refuse].

For regression analyses evaluating association between predictors and outcome, we identified three domains of interest: place of death, healthcare utilisation, and formal and informal costs in the last year of life.^2 3^

To determine place of death TILDA asks, “Where did [the person] die?”, with nine potential responses: own home; other person's home; hospital; inpatient hospice; nursing home; residential home; mixed nursing/residential home; sheltered housing; other place specified by interviewee.

TILDA asks about formal utilisation of public and private services across all major domains (e.g. hospital, general practitioner (GP), nursing home and residential care, home care, allied health) in the last year of life. Informal care is measured as number of hours’ unpaid help received in managing activities of daily living (ADL)^4^ and instrumental activities of daily living (IADL)^5^ in the last three months of life. These deficits are identified by asking which activities the decedent required help with: dressing, crossing a room, bathing, eating, getting in/out of bed, toileting (ADLs); preparing hot meals, household chores, shopping for groceries, making telephone calls, taking medication, managing money (IADLs).

We analysed both frequency utilisation and associated costs. For frequency utilisation, we identified five domains based on international literature ^2 6^ and prior analyses in TILDA^7-9^: hospital inpatient, outpatient and emergency department (ED) visits; GP visits; and formal home supports (at least one of home help, meals on wheels, personal care attendant and/or public health nurse).

To estimate costs we combined unit costs for health and social care use among the older population in Ireland with recorded utilisation.^10^ For informal costs in the last year of life, we combined end-of-life respondent data on unpaid help in the last three months, and nine months’ unpaid help from the last regular wave before death. Informal care costs were capped at a maximum of 16 hours per day for a participant with one carer and 24 hours per day for two or more carers.

#### Explanatory variables

TILDA collects a wide range of demographic, socioeconomic and health status data on participants. To identify explanatory variables we drew on Andersen’s model of predisposing, enabling, need characteristics; and prior utilisation.^11^ Predisposing characteristics are individual propensities to access care that exist prior to onset of illness (e.g. age, gender). Enabling characteristics are “means” to use services (e.g. income, entitlements). Need characteristics reflect burden of illness (including diagnoses, level of incapacity). Healthcare use is prior utilisation of services.

For presence of serious chronic diseases we created separate variables for diagnosis of four leading causes of death (cancer, heart disease, lung disease, dementia; where multiple diagnoses are possible) as well as an overall burden variable totalling the presence of 17 conditions (see Table 1 in main manuscript).

We used an *ex post* binary variable ‘short illness’, taken from the end-of-life interview: was the person who died ill for less than a week prior to death? [==0 | 1] This question was asked independently of the questions on chronic diseases and therefore it was possible for a decedent to have had a diagnosis of (e.g.) cancer or heart disease for months or years prior to death. A value of 1 for ‘short illness’ was strongly correlated with ‘unexpected’ in response to another end-of-life interview variable that we did not use: “Was the death expected at about the time it occurred, or was it unexpected?”

We used a cumulative deficits approach to construct a frailty index (FI).^9 12^ FI deficits included symptoms, diseases, disabilities and laboratory abnormalities associated with age and adverse outcomes, present in at least 1% of the population, covering several organ systems and which had under 5% missing data.^13^ A 32-item index was constructed by summing the number of deficits and dividing this number by the total number of possible deficits. A binary measure indicated the presence of frailty using an established cut-point (“frail”: FI score ≥0.25).

A list of 23 explanatory variables was compiled that we hypothesised to be connected to outcomes.^2 6-8 14^ Variables were measured variously in the last three months, last year and the last two years of life:

- **Predisposing:** age at death (years); gender (female | male); living with (alone | spouse | others);
- **Enabling:** Location of residence (Dublin | other urban | rural); education level (primary | secondary | tertiary), medical card or GP card (0|1); private health insurance (0|1);
- **Need:** diagnosis of: cancer (0|1), dementia (0|1), heart disease (0|1), lung disease (0|1); total chronic disease burden; ADLs (0-6); IADLs (0-6); frailty (0|1; = 1 if FI score ≥0.25); end-of-life interviewee responses on length of illness, physical health, depression;
- **Prior use:** high use of hospital [= 1 if the participant had 1+ hospital admission in last year of life OR 3+ more visits in last two years of life, excluding an admission ending in death], hospice [=1 if the participant EITHER lived for 3+ months and died there, OR entered the relevant setting to live and was discharged alive], nursing home [=1 if the participant EITHER lived for 3+ months and died there, OR entered the relevant setting to live and was discharged alive], home care [==1 if accessed at least one of home help, meals on wheels, personal care attendant and/or public health nurse in last year of life] and informal care [= 1 if 1<= hours help daily].

#### Model development

To promote model parsimony, we binarised multi-level variables and aggregated presence-of-disease variables (end-of-life interview responses and participant responses in regular waves) where supported by the Akaike Information Criterion (AIC).^15^ Using the designated groups predisposing, enabling, need and prior utilisation we ran four separate regressions to assess each variable’s potential link to outcomes of interest. Nineteen variables with a non-negligible (p<0.25) relationship were retained and four variables with (p<0.25) for all places of death were excluded: IADL total, physical health and depression per the end-of-life interview, and lung disease. A single, 19-variable model for place of death failed the Copas test for overfitting.^14^ We ran a series of comparative evaluations using the –fitstat- command in Stata, finalising a model with 14 predictors that are listed in Table 1 of the main manuscript. We evaluated need variables for signs of collinearity and were satisfied that this risk was low. All five prior utilisation variables were excluded using –fitstat- despite previously reported association with place of death.^6^

### 3 How analytic sample was reached

Appendix Figure 1 illustrates how the analytic sample was reached: 8,504 people took part in the first wave of TILDA, 7,610 in the second wave in 2012, and 6,566 in the third wave in 2014. Of 516 confirmed deaths to Wave 3, 375 (73%) had an end-of-life interview completed by a friend or family member.

An end-of-life response rate including those for whom an interview was possible (i.e. excluding from the denominator also those for whom an interview will be sought in the following wave) for TILDA is approximately 83%.^16^

<Appendix Figure 1 here>

Legend to Figure 1: Of the 25 W2>3 questionnaires collected from W1>2 dropouts, seven died in W1>W2 but follow-up occurred too late for inclusion in first EOL cohort.

### 4 Comparison of decedents with and without interviews

Per Appendix Figure 1, there have been 516 confirmed deaths for TILDA participants between Waves 1 and 3. Also as described in Figure 1, a small number of participants were recruited in Wave 2. Of the 516 deaths, family or close friends provided end-of-life interviews for 375 decedents. Of these 375 decedents for whom an exit interview was recorded, 366 were recruited in Wave 1 and nine in Wave 2. Of the 141 decedents for whom an end-of-life interview was not recorded, 134 were recruited in Wave 1 and seven in Wave 2.

To evaluate differences between those for whom an interview was completed and those not, we assessed descriptors at Wave 1. This therefore excluded 16 decedents who were recruited at Wave 2: nine for whom an end-of-life interview was recorded, and seven for whom it was not.

At recruitment, data were collected on 13 of the 14 variables that we include in our primary analyses (the exception is ‘short illness’, i.e. how long was the decedent ill prior to death?). We therefore compared those decedents who had been recruited at Wave 1 and for whom an interview was later completed (n=366) and those for whom an interview was not completed (n=134) on these 13 variables at baseline.

We used bivariate logistic regression and t-tests to test for difference on binary and continuous variables respectively (Appendix Table 1), and we performed a multivariate logit with a binary outcome of interest (did participant have an end-of-life interview?) and all 13 predictors (Appendix Table 2).

One association is significant in both analyses – those with an interview were significantly older (average 75.3 years at Wave 1) than those without (72.0). One possible interpretation is that the non-interview deaths were more sudden and so respondents were more reluctant to participate, although this is not reflected in significantly higher disability or illness burden among decedents with an interview.

Appendix Table 1 Bivariate comparison of those with and without interviews (N=500)

| **End-of-life Questionnaire?** | **Yes (N=366)** | **No (N=134)** | **P value** |
| --- | --- | --- | --- |
| **Gender:** Female | 46% | 43% | 0.64 |
| **Living alone:** Yes | 43% | 42% | 0.83 |
| **Education** Tertiary/higher | 15% | 23% | **0.04** |
| **Residence:** A rural area | 47% | 51% | 0.39 |
| **Medical or GP card:** Yes | 84% | 81% | 0.39 |
| **Private health insurance:** Yes | 38% | 41% | 0.51 |
| **Diagnosis of:** Heart disease | 26% | 22% | 0.32 |
| **Diagnosis of:** Cancer | 16% | 13% | 0.51 |
| **Diagnosis of:** Dementia | 1% | 1% | 0.72 |
| **Frailty:** Yes | 38% | 36% | 0.70 |
| **Age:** Mean years (SD) | 75.3 (10.4) | 72.0 (11.1) | **<0.01** |
| **ADL:** Mean total (SD) | 0.48 (1.11) | 0.43 (1.05) | 0.63 |
| **Chronic conditions:** Mean total (SD) | 0.53 (1.31) | 0.43 (1.26) | 0.44 |

All data at Wave 1. SD: Standard deviation. For full Legend see Table 1 in main manuscript.

Appendix Table 2 Multivariate associations with having an EOL interview (versus not having one) (N=500)

|  | **Coefficient** | **P value** | **95%** | **CI** |
| --- | --- | --- | --- | --- |
| **Gender:** Female | 0.08 | 0.73 | -0.35 | 0.50 |
| **Living alone:** Yes | -0.01 | 0.95 | -0.43 | 0.41 |
| **Education** Tertiary/higher | -0.52 | 0.07 | -1.08 | 0.04 |
| **Residence:** A rural area | -0.30 | 0.16 | -0.71 | 0.11 |
| **Medical or GP card:** Yes | -0.40 | 0.22 | -1.04 | 0.24 |
| **Private health insurance:** Yes | -0.11 | 0.66 | -0.59 | 0.37 |
| **Diagnosis of:** Heart disease | 0.20 | 0.45 | -0.32 | 0.73 |
| **Diagnosis of:** Cancer | 0.34 | 0.27 | -0.26 | 0.94 |
| **Diagnosis of:** Dementia | -0.49 | 0.59 | -2.28 | 1.29 |
| **Frailty:** Yes | -0.22 | 0.41 | -0.74 | 0.30 |
| **Age:** Mean years (SD) | **0.04** | **<0.01** | **0.01** | **0.06** |
| **ADL:** Mean total (SD) | 0.01 | 0.93 | -0.21 | 0.22 |
| **Chronic conditions:** Mean total (SD) | 0.07 | 0.46 | -0.11 | 0.25 |

### 5 Comparison of decedents with interviews and the general population

Deaths in TILDA waves 1-3 occurred 2010-2014. The Irish Central Statistics Office issues a ‘Vital Statistics’ summary of births and deaths each year. To evaluate differences between our sample (N=375) and the general population of decedents age 50+ in 2012 (mid-point of the analytic period), we compared age at death and cause of death in Appendix Table 3. TILDA deaths were on average slightly younger and with a higher proportion of cancer deaths.

Appendix Table 3 Comparison of sample and population level decedents aged 50+

|  | **Sample (N=375)** | **Population (N=26,849)** |
| --- | --- | --- |
| **Age at death:** Mean years | 77.7 | 78.4 |
| **Cause of death:** Cancer | 38% | 30% |
| **Cause of death:** CHF | 30% | 25% |

### 6 Sensitivity analysis

To affirm our key conclusions from the place of death analyses in the main manuscript, we performed two sensitivity analyses.

First we checked robustness of our results to low cell counts: we cross-tabulated our four outcomes of interest by all predictors (Appendix Table 4).

Appendix Table 4 Outcomes-by-predictors cross-tabulation

|  | **Place of death** | **Hospital (n=172)** | | **Home**  **(n=100)** | | **Hospice**  **(n=43)** | | **Nursing Home**  **(n=39)** | |
| --- | --- | --- | --- | --- | --- | --- | --- | --- | --- |
|  |  | ***N*** | ***%*** | ***N*** | ***%*** | ***N*** | ***%*** | ***N*** | ***%*** |
| **Gender** | *Female* | 82 | 48% | 40 | 40% | 19 | 44% | 18 | 44% |
| **Living alone** | *Yes* | 78 | 45% | 33 | 33% | 16 | 37% | 25 | 61% |
| **Education** | *Tertiary/higher* | 23 | 13% | 17 | 17% | 13 | 30% | 2 | 5% |
| **Location** | *Rural* | 89 | 52% | 49 | 49% | 9 | 21% | 16 | 39% |
| **Medical/GP card** | *Yes* | 148 | 86% | 87 | 87% | 38 | 88% | 40 | 98% |
| **Insurance** | *Yes* | 55 | 32% | 40 | 40% | 21 | 49% | 12 | 29% |
|  |  |  |  |  |  |  |  |  |  |
| **Diagnosis of** | *Heart disease* | 92 | 53% | 61 | 61% | 11 | 26% | 26 | 63% |
|  | *Cancer* | 68 | 40% | 47 | 47% | 40 | 93% | 5 | 12% |
|  | *Dementia* | 16 | 9% | 8 | 8% | 2 | 5% | 21 | 51% |
| **Short illness** | *Yes* | 28 | 16% | 35 | 35% | 1 | 2% | 2 | 5% |
| **Frailty** | *Yes* | 65 | 38% | 35 | 35% | 16 | 37% | 17 | 41% |
|  |  |  |  |  |  |  |  |  |  |
|  |  | ***Mean*** | ***SD*** | ***Mean*** | ***SD*** | ***Mean*** | ***SD*** | ***Mean*** | ***SD*** |
| **Age** | *Years* | 77.8 | 10.1 | 76.7 | 11.0 | 73.7 | 8.2 | 86.1 | 7.7 |
| **ADL** | *Total (/6)* | 2.1 | 2.4 | 2.0 | 2.6 | 2.9 | 2.8 | 4.8 | 1.9 |
| **Chronic conditions** | *Total (/17)* | 2.8 | 1.6 | 2.6 | 1.6 | 3.3 | 1.9 | 3.3 | 2.1 |

SD: Standard deviation

For continuous variables (age, ADL total, chronic condition total) we are not concerned about imbalance (e.g. near absence of ADLs in people dying in one location).

For binary variables there are seven cells where cell count is low (<10): dementia/home; rural/hospice; dementia/hospice; short illness/hospice; education/nursing home; cancer/nursing home; short illness/nursing home.

In each case we re-ran the regression without the predictor in question; in each case the significant associations reported in the primary analysis for the place of death in question are unchanged (data not shown).

Second, we repeated our regressions with additional variables. Specifically, we added binary variables for high prior use of specific services, which have been found to be strongly associated with place of death:^6^

- High hospital use (= 1 if the participant had 1+ hospital admission in last year of life OR 3+ more visits in last two years of life, excluding an admission ending in death)
- High nursing home and hospice use (=1 if the participant EITHER lived for 3+ months in the relevant setting OR entered the relevant setting to live and was discharged alive).
- Home care: accessed at least one of home help, meals on wheels, personal care attendant and/or public health nurse in last year of life.
- Informal care: at least one hour daily unpaid support from family/friends.

The results are given in Appendix Table 4:

- Statistically significant associations for home and hospice death versus hospital death are wholly consistent with our primary analyses.
- Statistically significant associations for nursing home death differ as follows: age (p=0.07), rural residency (0.08) and cancer condition (0.25) are no longer significant, and the additional variable prior nursing home use (p<0.01) is. Given that (a) age, rural residency and cancer are all significantly associated with nursing home use; (b) in two of the three cases the p value is close to 0.05; (c) our model evaluation identified the model without utilisation variables as superior; and (d) potential endogeneity concerns; we are satisfied that the results reported in the primary analysis are substantively supported.

### 7 Comparison of decedents on ‘short illness’

In the primary analyses, our *ex post* variable ‘short illness’ (was the person who died ill for less than a week prior to death?) consistently exhibits statistically significant associations with our outcomes of interest. This reflects heterogeneity within a relatively young sample of decedents and suggests the potential for more than one latent class with different distributions of utilisation and determinants of outcome. Some of our results differ from prior TILDA analyses,^7-9^ e.g. negative associations between age and frailty, and outpatient utilisation, and it is possible that larger future samples will show more distinct classes with different determinants of outcome.

To check the health status of participants from their own perspective against the response of the end-of-life interviewee, we extracted age, diagnosis, frailty and ADL data in each participant’s last interview prior to death and compared for those who died following a ‘short illness’ and not. The results are in Appendix Table 6. Those who died following a ‘short illness’ were on average younger, with a lower prevalence of all three life-limiting conditions and of frailty, lower mean ADL total but higher total number of conditions. Two associations are statistically significant: cancer and ADL total.

Appendix Table 5 Sensitivity analysis for place of death analyses

|  |  | **Home** | | | | **Inpatient hospice** | | | | **Nursing Home** | | | |
| --- | --- | --- | --- | --- | --- | --- | --- | --- | --- | --- | --- | --- | --- |
|  |  | **RRR** | **p** | **95% CI** | | **RRR** | **p** | **95% CI** | | **RRR** | **p** | **95% CI** | |
| **Age** | *Years* | 0.99 | 0.52 | 0.96 | 1.02 | 0.99 | 0.56 | 0.94 | 1.03 | 1.07 | 0.07 | 1.00 | 1.15 |
| **Gender** | *Female* | 0.71 | 0.24 | 0.41 | 1.26 | 0.66 | 0.34 | 0.29 | 1.53 | 0.58 | 0.32 | 0.20 | 1.69 |
| **Living alone** | *Yes* | **0.52** | **0.02** | **0.29** | **0.91** | 0.62 | 0.27 | 0.27 | 1.44 | 1.87 | 0.28 | 0.60 | 5.80 |
| **Education** | *Tertiary/higher* | 1.24 | 0.59 | 0.56 | 2.75 | 1.64 | 0.35 | 0.59 | 4.56 | 0.14 | 0.09 | 0.01 | 1.39 |
| **Location** | *Rural* | 1.01 | 0.98 | 0.58 | 1.75 | 0.43 | 0.08 | 0.17 | 1.09 | 0.38 | 0.08 | 0.13 | 1.13 |
| **Medical/GP card** | *Yes* | 1.62 | 0.29 | 0.66 | 3.95 | 1.54 | 0.51 | 0.43 | 5.58 | 0.98 | 0.99 | 0.09 | 10.54 |
| **Insurance** | *Yes* | 1.29 | 0.43 | 0.69 | 2.39 | 1.56 | 0.34 | 0.63 | 3.86 | 1.73 | 0.36 | 0.54 | 5.59 |
| **ADL** | *Total/6* | 1.06 | 0.43 | 0.92 | 1.21 | 1.06 | 0.50 | 0.89 | 1.27 | **1.53** | **<0.01** | **1.18** | **1.99** |
| **Diagnosis of** | *Heart disease* | 1.35 | 0.39 | 0.68 | 2.67 | **0.24** | **0.01** | **0.08** | **0.73** | 0.75 | 0.64 | 0.22 | 2.56 |
|  | *Cancer* | **2.43** | **0.01** | **1.24** | **4.75** | **8.67** | **<0.01** | **2.24** | **33.56** | 0.43 | 0.25 | 0.10 | 1.81 |
|  | *Dementia* | 1.06 | 0.92 | 0.37 | 2.99 | 0.80 | 0.82 | 0.12 | 5.16 | **3.77** | **0.04** | **1.04** | **13.65** |
| **Chronic conditions** | *Total* | 0.91 | 0.35 | 0.74 | 1.11 | **1.48** | **0.02** | **1.07** | **2.04** | 0.99 | 0.95 | 0.68 | 1.44 |
| **Short illness** | *Yes* | **4.09** | **<0.01** | **1.82** | **9.18** | 1.09 | 0.94 | 0.11 | 10.64 | 0.25 | 0.19 | 0.03 | 1.95 |
| **Frailty** | *Yes* | 1.09 | 0.81 | 0.55 | 2.14 | 0.79 | 0.68 | 0.27 | 2.34 | 0.50 | 0.26 | 0.15 | 1.67 |
| **Hospital use** | *Yes* | 0.55 | 0.05 | 0.30 | 1.00 | 1.00 | 1.00 | 0.41 | 2.46 | 0.48 | 0.20 | 0.16 | 1.49 |
| **Hospice use** | *Yes* | dropped | | | | 7.04 | 0.12 | 0.61 | 81.24 | dropped | | | |
| **Nursing home use** | *Yes* | 0.78 | 0.69 | 0.24 | 2.55 | 1.70 | 0.58 | 0.27 | 10.90 | **5.87** | **<0.01** | **1.90** | **18.18** |
| **Home care use** | *Yes* | 2.00 | 0.05 | 1.02 | 3.93 | 1.73 | 0.26 | 0.67 | 4.50 | 0.56 | 0.32 | 0.18 | 1.76 |
| **Informal carer** | *Yes* | 0.99 | 0.99 | 0.49 | 2.00 | 1.61 | 0.37 | 0.57 | 4.55 | 0.37 | 0.11 | 0.11 | 1.24 |

For variables legend, see Table 1 in main manuscript. p: p value. RRR: Relative risk ratio; 95% CI: confidence interval. Statistically significant (p<0.05) highlighted bold.

Appendix Table 6 Bivariate comparison of those who died after a short illness (and not)

| **Short illness?** | **Yes (N=75)** | **No (N=300)** | **P value** |
| --- | --- | --- | --- |
| **Diagnosis of:** Heart disease | 28% | 32% | 0.56 |
| **Diagnosis of:** Cancer | 11% | 23% | **0.02** |
| **Diagnosis of:** Dementia | 4% | 7% | 0.34 |
| **Frailty:** Yes | 34% | 45% | 0.10 |
| **Age:** Mean years (SD) | 76.2 (10.2) | 78.0 (10.2) | 0.18 |
| **ADL:** Mean total (SD) | 0.37 (1.10) | 0.92 (1.70) | **0.008** |
| **Chronic conditions:** Mean total (SD) | 0.76 (1.59) | 0.47 (1.22) | 0.09 |

All data from last participant interview prior to death

### References

1. Donoghue O, Foley M, Kenny RA. Cohort Maintenance Strategies Used by The Irish Longitudinal Study on Ageing. Dublin: TILDA, 2017.

2. Langton JM, Blanch B, Drew AK, et al. Retrospective studies of end-of-life resource utilization and costs in cancer care using health administrative data: a systematic review. *Palliative medicine* 2014;28(10):1167-96. doi: 10.1177/0269216314533813 [published Online First: 2014/05/29]

3. Bekelman JE, Halpern SD, Blankart CR, et al. Comparison of Site of Death, Health Care Utilization, and Hospital Expenditures for Patients Dying With Cancer in 7 Developed Countries. *JAMA : the journal of the American Medical Association* 2016;315(3):272-83. doi: 10.1001/jama.2015.18603 [published Online First: 2016/01/20]

4. Katz S, Ford A, Moskowitz R, et al. The index of ADL: a standardized measure of biological and psychological function. *JAMA : the journal of the American Medical Association* 1963;185(12):914-19.

5. Lawton MP, Brody EM. Assessment of older people: self-maintaining and instrumental activities of daily living. *The Gerontologist* 1969;9(3):179-86.

6. Costa V, Earle CC, Esplen MJ, et al. The determinants of home and nursing home death: a systematic review and meta-analysis. *BMC palliative care* 2016;15:8. doi: 10.1186/s12904-016-0077-8 [published Online First: 2016/01/23]

7. Nolan A, Ma Y, Moore P. Changes in Public Healthcare Entitlement and Healthcare Utilisation among the Older Population in Ireland. Dublin: The Irish Longitudinal Study on Ageing, 2016.

8. McNamara A, Normand C, Whelan B. Patterns and Determinants of Health Care Utilisation in Ireland. Dublin: The Irish Longitudinal Study on Ageing (TILDA), 2013.

9. Roe L, Normand C, Wren MA, et al. The impact of frailty on healthcare utilisation in Ireland: evidence from the Irish longitudinal study on ageing. *BMC geriatrics* 2017;17(1):203. doi: 10.1186/s12877-017-0579-0 [published Online First: 2017/09/07]

10. Brick A, Normand C, O'Hara S, et al. Economic Evaluation of Palliative Care in Ireland: Final report: Trinity College Dublin; 2015 [Report prepared for The Atlantic Philanthropies]. Available from: <http://www.medicine.tcd.ie/health_policy_management/assets/pdf/Final-report-July-2015.pdf>.

11. Andersen RM. Revisiting the behavioral model and access to medical care: does it matter? *Journal of health and social behavior* 1995;36(1):1-10. [published Online First: 1995/03/01]

12. Rockwood K, Song X, MacKnight C, et al. A global clinical measure of fitness and frailty in elderly people. *CMAJ : Canadian Medical Association Journal* 2005;173(5):489-95. doi: 10.1503/cmaj.050051

13. Searle SD, Mitnitski A, Gahbauer EA, et al. A standard procedure for creating a frailty index. *BMC geriatrics* 2008;8(1):24. doi: 10.1186/1471-2318-8-24

14. Copas JB. Regression, Prediction and Shrinkage. *Journal of the Royal Statistical Society Series B (Methodological)* 1983;45(3):311-54. doi: 10.2307/2345402

15. Burnham KP, Anderson DR. Multimodel Inference: Understanding AIC and BIC in Model Selection. *Sociological Methods & Research* 2004;33(2):261-304. doi: 10.1177/0049124104268644

16. Donoghue OA, McGarrigle CA, Foley M, et al. Cohort Profile Update: The Irish Longitudinal Study on Ageing (TILDA). *International Journal of Epidemiology* 2018;47(5):1398-98l. doi: 10.1093/ije/dyy163
